# Supplementary material for: Gentrification, measures of neighborhood change, and infant mortality in Michigan
Source: Res Sq. 2024 Nov 11:rs.3.rs-5306929. Preprint. [Version 1] doi: 10.21203/rs.3.rs-5306929/v1 (PMC11601844; doi:10.21203/rs.3.rs-5306929/v1)
Supplement: Supplement 1 [file NIHPPRS5306929V1-supplement-1.pdf]

Supplementary Files

This is a list of supplementary files associated with this preprint. Click to download.

- AppendixTable10930.docx
- Appendix20930.docx
- Appendix30930.docx
